# Supplementary material for: Habituation and individual variation in the endocrine stress response in the Trinidadian guppy (Poecilia reticulata)
Source: Gen Comp Endocrinol. 2019 Jan 1;270:113–22. doi: 10.1016/j.ygcen.2018.10.013 (PMC6300406; doi:10.1016/j.ygcen.2018.10.013)
Supplement: Supplementary Data 1 — Appendix A: Analysis of conjugated fractions of cortisol and 11-ketotestosterone. [file mmc1.docx]

**Appendix: Analysis of conjugated fractions of cortisol and 11-ketotestosterone**

*Introduction & methods*

In this study, we separated both target hormones (cortisol and 11-ketotestosterone, 11KT) into their ‘free’ and ‘conjugated’ fractions for analysis. We present results of our analysis of free hormones in the main text, as the concentration of free hormone in the water is taken to scale with the ‘physiologically active’ concentration of hormones in the fish’s circulation across the duration of the sampling period. The concentration of conjugated hormone in the water is thought to reflect the concentration of hormone in the fish’s circulation over a greater time lag extending to the period prior to exposure to the stressor (Scott and Ellis, 2007). In this appendix, we present results of our tests for repeat effects on the conjugated form (and for relationships between these fractions) as these may provide further evidence of individual variation in the ability to habituate to a repeated stressor. We anticipate that conjugated forms should show a time-lagged effect, such that there would be a shift in the conjugated “habituation” curve for conjugated relative to free cortisol (i.e., an initial increase at the second exposure to the stressor, followed by a lagged decrease). We have no specific prediction for conjugated 11KT other than that it should show a time-lagged version of the free pattern. Note that all analyses are performed as described in the main text for the free hormones.

*Results*

We find significant effects of repeated stress exposure on conjugated hormone fractions. For the conjugated form of cortisol this appears to be due largely to increased concentrations at exposures 3 and 4 relative to earlier exposures (Table A1a). We find no sex differences in how these levels changed over the repeated measures (sex × stressor number interaction: F_3, 62.9_ = 0.91, *P* = 0.44). We also find no clear sex differences in mean conjugated cortisol concentrations, although there is a positive effect of mass. Concentrations of the conjugated form of 11KT also change over repeats, rising to a peak at the third exposure before decreasing in the final exposure (Table A1b). Males exhibit consistently higher levels of conjugated 11KT, and there are no sex differences in the response to repeated assays (F_3, 88.7_ = 0.86, *P* = 0.46).

Under the random regression approach, there is significant variance among individuals in the conjugated forms of both hormones. We find moderate adjusted repeatability for conjugated cortisol (R=0.26 SE 0.10, χ^2^_0,1_ = 8.91, *P* = 0.001) and for 11KT (R=0.27 (SE 0.10), χ^2^_0,1_ = 9.30, *P* = 0.001). For conjugated cortisol the comparison of random intercept and random slope models is non-significant (χ^2^_2_ = 1.64, *P* = 0.44). For conjugated 11KT we do not detect any estimable variance in random slope (χ^2^_2_ = 0, *P* = 1).

Using the character state approach, conjugated cortisol shows no significant change in variance over repeats, but there is significant within-individual covariance structure. The covariance-correlation matrix shows that correlations between pairs of assays are weakly positive (ranging from 0.131 to 0.411, Table A2a), and we also find a qualitative pattern of declining strength as the inter-observation time increases. Conjugated 11KT does show changes in variance across repeats (χ^2^_3_ = 33.3, *P* < 0.001), as well as within-individual covariance structure (χ^2^_6_ = 19.8, *P* = 0.003) and a pattern of positive correlations among repeats (ranging from 0.124 to 0.561, Table A2b), which tend to decline in strength with increasing inter-observation interval.

*Discussion*

While the conjugated forms of the hormones do change over repeated exposures, these do not appear to track free hormones in the simple time-lagged manner we had expected. Unlike free circulating cortisol, the conjugated form of this hormone shows no strong sex differences, and instead shows a linear increase with body mass (discussed further in the main text). We find no evidence for among-individual variation in patterns of habituation in either conjugated cortisol or 11KT, although both show moderate among-individual variance in mean response.

The conjugated forms of both cortisol and 11KT peaked at the third repeat; while this could conceivably show the effects of stress on a longer timescale in cortisol, the fact that conjugated 11KT shows a similar pattern despite very different responses in free hormones undermines this claim somewhat. Intriguingly, conjugated forms of both cortisol and 11KT also show similar patterns of individual variation: significant covariance structure indicates repeatability, and measurements made closer together in time tend to be more strongly (positively) correlated than those further apart. Reaction norm models (being a reduced-rank form of the above) largely ratified these results, with significant repeatability found in both conjugated hormones. One possible explanation might be that variation in conjugated hormones is explained to some extent by differences in steroid metabolism, for example in the rates of conjugating enzyme production.

| Hormone | Effect | Coefficient (SE) | DF | F | P |
| --- | --- | --- | --- | --- | --- |
| Cortisol (conjugated) | (Intercept) | -1.125 (0.146) | 1,26.9 | 94.7 | <0.001 |
|  | Assay 1 | 0 (-) | 3,64.3 | 3.3 | 0.026 |
|  | Assay 2 | -0.035 (0.105) |  |  |  |
|  | Assay 3 | 0.277 (0. 110) |  |  |  |
|  | Assay 4 | 0.105 (0. 117) |  |  |  |
|  | Sex (Male) | 0.207 (0. 205) | 1,27.1 | 1.0 | 0.322 |
|  | Body mass | 0.557 (0.103) | 1,27.2 | 29.3 | <0.001 |
|  | Order | 0.010 (0.010) | 1,115.6 | 1.1 | 0.295 |
|  | Tank A | 0 (-) | 1,26.9 | 2.4 | 0.132 |
|  | Tank B | -0.186 (0.119) |  |  |  |
| 11-ketotestosterone (conjugated) | (Intercept) | 1.764 (0.163) | 1,27.3 | 217.1 | <0.001 |
|  | Assay 1 | 0 (-) | 3,91.1 | 9.8 | <0.001 |
|  | Assay 2 | 0.132 (0.126) |  |  |  |
|  | Assay 3 | 0.646 (0. 126) |  |  |  |
|  | Assay 4 | 0.176 (0. 126) |  |  |  |
|  | Sex (Male) | 1.381 (0.241) | 1,27.4 | 32.9 | <0.001 |
|  | Body mass | 0.165 (0.120) | 1,27.4 | 1.9 | 0.182 |
|  | Order | 0.037 (0.012) | 1,118.0 | 10.2 | 0.002 |
|  | Tank A | 0 (-) | 1,27.4 | 1.3 | 0.269 |
|  | Tank B | -0.158 (0.140) |  |  |  |

Table A1: Fixed effect estimates from mixed-effects model analyses of conjugated (a) cortisol and (b) 11-ketotestosterone levels in individual guppies over four repeated measures.

| c) Conjugated cortisol | **Repeat 1** | **Repeat 2** | **Repeat 3** | **Repeat 4** |
| --- | --- | --- | --- | --- |
| **Repeat 1** | 0.238 (0.116,0.367) | 0.411 (-0.008,0.519) | 0.202 (-0.394,0.356) | 0.131 (-0.499,0.327) |
| **Repeat 2** | 0.126 (-0.001,0.245) | 0.396 (0.198,0.609) | 0.379 (-0.04,0.492) | 0.162 (-0.376,0.368) |
| **Repeat 3** | 0.045 (-0.042,0.122) | 0.109 (-0.005,0.217) | 0.208 (0.097,0.321) | 0.367 (-0.052,0.508) |
| **Repeat 4** | 0.029 (-0.053,0.11) | 0.046 (-0.052,0.16) | 0.075 (-0.005,0.16) | 0.201 (0.098,0.309) |

| d) Conjugated 11KT | **Repeat 1** | **Repeat 2** | **Repeat 3** | **Repeat 4** |
| --- | --- | --- | --- | --- |
| **Repeat 1** | 0.231 (0.105,0.346) | 0.477 (0.113,0.582) | 0.176 (-0.404,0.371) | 0.124 (-0.6,0.31) |
| **Repeat 2** | 0.157 (0.018,0.292) | 0.471 (0.246,0.729) | 0.267 (-0.184,0.439) | 0.372 (-0.034,0.517) |
| **Repeat 3** | 0.1 (-0.105,0.313) | 0.215 (-0.074,0.537) | 1.381 (0.646,2.053) | 0.561 (0.273,0.645) |
| **Repeat 4** | 0.029 (-0.062,0.109) | 0.123 (-0.005,0.264) | 0.319 (0.07,0.553) | 0.233 (0.102,0.357) |

Table A2: Covariance-correlation matrix (conditional on fixed effects) for conjugated circulating (a) cortisol, (b) 11KT. Variances are on shaded diagonals, covariances below and correlations above. In parentheses are the 95% confidence intervals on calculated from 5000 bootstrapped replicates.
