# Supplementary material for: Habituation and individual variation in the endocrine stress response in the Trinidadian guppy (Poecilia reticulata)
Source: Gen Comp Endocrinol. 2019 Jan 1;270:113–22. doi: 10.1016/j.ygcen.2018.10.013 (PMC6300406; doi:10.1016/j.ygcen.2018.10.013)
Supplement: Supplementary Data 3 — Appendix C: R code for analyses. [file mmc3.pdf]

Individual variation in stress habituation in the Trinidadian guppy: Supplemental R code

Tom Houslay

5/30/2018

Habituation in stress hormones

Below is code used in our manuscript, "Individual variation in stress habituation in the Trinidadian guppy (*Poecilia reticulata*)". The statistical analysis was performed in `R` version 3.4.1, and largely uses the proprietary software `ASreml`. Note that similar modelling can also be performed in a Bayesian framework in R using the free package `MCMCglmm`, and we have provided tutorials on the use of this for multivariate modelling linked to an earlier paper (see tomhouslay.com/tutorials).

Libraries and custom functions

We need to load libraries (these may have to be installed through your `R` IDE), and custom functions that are to be used later in the script.

```
library(knitr)
library(tidyverse)
library(stringr)

library(mvtnorm)
library(coda)

library(asreml)
library(nadiv)

# Standard error function
stderr <- function(x) {
  x <- x[!is.na(x)]
  se <- sd(x)/sqrt(length(x))
  return(se)
  }
```

A number of these functions are specifically for dealing with the matrix-based methods of this paper, including converting the variance components of an `ASreml` object into a matrix, performing bootstrapping routines on a matrix, etc:

```
# BOOTSRAPPING FUNCTION
# - ARGUMENTS
#  -- asreml model object
#  -- number of traits
# - RETURNS
#  -- matrix of 5000 bootstrap replicates of the covariance matrix from the model

##
# NOTE:
#  - This is for the residual covariance matrix,
#      if using other parts of the variance components then edit as necessary
```

```
bootRepMat <- function(asr_model, n) {

  # Get the number of variances and covariances in the diagonal matrix
  diag_size <- 0.5*(n*(n+1))

  # Extract variance components of interest and create data frame of these values
  # NOTE needs to filter on required set
  model_df <- data_frame(Var = row.names(summary(asr_model)$varcomp),
                         Num = summary(asr_model)$varcomp$component) %>%
    filter(str_sub(Var, 1, 16) == "Replicate_fac:ID")


  # Average information
  model_ai <- as.numeric(asr_model$ai)
  # Sampling (co)-variances
  model_VC <- aiFun(asr_model, model_ai)

  # Subset sampling covariance matrix for useful parts
  # NOTE needs to subset appropriately
  model_VC <- model_VC[1:(diag_size),1:(diag_size)]


  # Create matrix
  model_mat <- vecToMat(model_df$Num, n)  ## Second value is number of traits
  # Get estimates as a vector
  model_ests <- model_mat[upper.tri(model_mat, diag=TRUE)]

  # Generate 5000 random draws from multivariate normal distribution
  #  with given means and covariances
  boot_matrix <- rmvnorm(5000, model_ests, model_VC)

  return(boot_matrix)

}
```

```
## Quick function for converting vector to symmetric matrix
vecToMat <- function(X, n) {
  S <- diag(n)
  S[upper.tri(S, diag=TRUE)] <- X
  S <- S + t(S) - diag(diag(S))
  return(S)
}

## Get 'Replicate_fac' matrix from an asreml object
getRepMat <- function(asr_model, n) {

  # Extract variance components
  model_df <- data_frame(Var = row.names(summary(asr_model)$varcomp),
                         Num = summary(asr_model)$varcomp$component)

  model_df <- model_df %>%
    filter(str_sub(Var, 1, 16) == "Replicate_fac:ID")
```

```r
  return(vecToMat(model_df$Num, n))  ## Second value is number of traits

}

## Get nice version of 'Replicate_fac' matrix for outputting:
# - includes bootstrapped CIs
# - correlations above and covariances below diagonal
getOutputMat_Rep <- function(asr_model, n){

  mat <- getRepMat(asr_model, n)
  bootmat <- bootRepMat(asr_model, n)

  CIlower <- vecToMat(as.numeric(HPDinterval(as.mcmc(bootmat), prob=0.95)[,'lower']),n)
  CIupper <- vecToMat(as.numeric(HPDinterval(as.mcmc(bootmat), prob=0.95)[,'upper']),n)

  mat_cor <- cov2cor(mat)
  CIlower_cor <- cov2cor(CIlower)
  CIupper_cor <- cov2cor(CIupper)


  mat_CI <- matrix(NA, n, n)

  for(i in 1:n){
    for(j in 1:n){
      if(j > i){
        mat_CI[i,j] <- paste(round(mat_cor[i,j],digits=3),
                                              " (",
                                              round(CIlower_cor[i,j],digits=3),
                                              ",",
                                              round(CIupper_cor[i,j],digits=3),
                                              ")",
                                              sep = "")
      }
      else{

        mat_CI[i,j] <- paste(round(mat[i,j],digits=3),
                                              " (",
                                              round(CIlower[i,j],digits=3),
                                              ",",
                                              round(CIupper[i,j],digits=3),
                                              ")",
                                              sep = "")
      }
    }
  }

  return(mat_CI)

}
```

Load data

Read in the file `pilot_hormones_conj.csv`. We then use some data wrangling to rename variables, or make some alterations – for example, we centre the `Replicate` variable manually below. Both free cortisol and 11KT were resuspended in 600uL of buffer, so we multiply their values by 0.6. Free cortisol was also diluted 1:32, so we multiply this value by 32. These new values give us the value of the whole sample. The conjugated versions were already calculated as the value for the sample, so are left unchanged from the data file.

```
df_hormones_read <- read_csv("pilot_hormones_conj.csv")

df_hormones <- df_hormones_read %>%
  mutate(Cort_ng = Cortisol_45*0.6*32,
         KT_pg = KT_75*0.6,
         Replicate_cen = Replicate - 2.5,
         Replicate_fac = factor(Replicate),
         Tank = ifelse(Tank %in% c(30,31),"A","B")) %>%
  select(ID, Tank, Replicate, Replicate_cen, Replicate_fac, SexM, Length, Mass, SampleOrder,
         Cort_ng, KT_pg,
         Cort_conj_ng = `Cortisol CONJ (ng/sample)`,
         KT_conj_pg = `KT CONJ (pg/sample)`)
```

Modelling data

Note that we provide here the 'final models' after simplification, rather than including all steps.

Habituation effects on mean hormone levels

Simplified fixed effects; random effect only of individual intercepts.

Free cortisol

```
asr_cort_fac_rr2 <- asreml(log(Cort_ng) ~ Replicate_fac + SexM +
                          scale(Mass) +
                          scale(SampleOrder, scale = FALSE) +
                            Tank,
                        random =~ str(~ID + ID:Replicate_cen, ~us(2):id(32)),
                        data = df_hormones)
```

```
wald(asr_cort_fac_rr2, denDF = "numeric", ssType = "conditional")
```

```
## ASReml: Wed May 30 12:56:13 2018
##
##      LogLik         S2      DF      wall     cpu
##      16.6780      0.1719   120  12:56:13     0.0
##      16.6780      0.1719   120  12:56:13     0.0
##      16.6780      0.1719   120  12:56:13     0.0
##      16.6780      0.1719   120  12:56:13     0.0
##
## Finished on: Wed May 30 12:56:13 2018
##
## LogLikelihood Converged
```

```
## $Wald
##                                  Df denDF     F.inc    F.con Margin
## (Intercept)                       1  27.3 29310.000 8845.000
## Replicate_fac                     3  64.7    14.220   14.220      A
## SexM                              1  27.5    37.800   22.560      A
## scale(Mass)                       1  27.5     2.053    2.042      A
## scale(SampleOrder, scale = FALSE)  1 100.0     2.574    2.533      A
## Tank                              1  27.3     2.456    2.456      A
##                                            Pr
## (Intercept)                       7.575981e-36
## Replicate_fac                     3.217677e-07
## SexM                              5.715868e-05
## scale(Mass)                       1.642348e-01
## scale(SampleOrder, scale = FALSE) 1.146539e-01
## Tank                              1.286007e-01
##
## $stratumVariances
## NULL
```

```
summary(asr_cort_fac_rr2, all = TRUE)$coef.fixed
```

```
##                                     solution   std error   z ratio
## Tank_A                             0.00000000          NA        NA
## Tank_B                             0.15305184 0.097664673  1.567116
## scale(SampleOrder, scale = FALSE) -0.01448774 0.009103186 -1.591502
## scale(Mass)                       -0.12021780 0.084122951 -1.429073
## SexM                              -0.79907972 0.168237164 -4.749722
## Replicate_fac_1                    0.00000000          NA        NA
## Replicate_fac_2                   -0.61704888 0.105597173 -5.843422
## Replicate_fac_3                   -0.57776164 0.111263528 -5.192732
## Replicate_fac_4                   -0.31775259 0.120114900 -2.645405
## (Intercept)                        8.95093226 0.116053735 77.127481
```

Free 11KT

```
asr_11kt_fac_rr0a <- asreml(log(KT_pg) ~ Replicate_fac * SexM +
                              scale(Mass) +
                              scale(SampleOrder, scale = FALSE) +
                              factor(Tank),
                            random =~ str(~ID + ID:Replicate_cen, ~us(2):id(32)),
                            data = df_hormones,
                            maxiter = 300)
```

```
wald.asreml(asr_11kt_fac_rr0a, denDF = "numeric", ssType = "conditional")
```

```
## ASReml: Wed May 30 12:56:13 2018
##
##  US matrix updates modified 1 times to remain positive definite.
##      LogLik         S2      DF      wall      cpu
##      79.9219      0.0653    117  12:56:13     0.0 (3 restrained)
##  US matrix updates modified 1 times to remain positive definite.
##      79.9229      0.0653    117  12:56:13     0.0 (3 restrained)
##  US matrix updates modified 1 times to remain positive definite.
##      79.9238      0.0653    117  12:56:13     0.0 (3 restrained)
##  US matrix updates modified 1 times to remain positive definite.
```

```
##      79.9247      0.0653    117  12:56:13     0.0 (3 restrained)
## US variance structures were modified in 4 instances to make them positive definite
##
## Finished on: Wed May 30 12:56:13 2018
##
## LogLikelihood Converged
```

```
## $Wald
##                                    Df denDF     F.inc     F.con Margin
## (Intercept)                         1  65.9 6192.0000 1178.0000
## Replicate_fac                       3  65.9   10.4700   10.4700      A
## SexM                                1  65.9  156.0000   71.6600      A
## scale(Mass)                         1  65.9    1.6330    2.1600      B
## scale(SampleOrder, scale = FALSE)   1  65.9    0.1388    0.3132      B
## factor(Tank)                        1  65.9    3.1080    3.1140      B
## Replicate_fac:SexM                  3  65.9    5.1110    5.1120      B
##                                             Pr
## (Intercept)                       9.523244e-44
## Replicate_fac                     1.014707e-05
## SexM                              3.958206e-12
## scale(Mass)                       1.464172e-01
## scale(SampleOrder, scale = FALSE) 5.775961e-01
## factor(Tank)                      8.228172e-02
## Replicate_fac:SexM                3.060126e-03
##
## $stratumVariances
## NULL
```

```
summary(asr_11kt_fac_rr0a, all = TRUE)$coef.fixed
```

```
##                                       solution   std error    z ratio
## Replicate_fac_1:SexM               0.000000000          NA         NA
## Replicate_fac_2:SexM              -0.402135075 0.128489650 -3.1297079
## Replicate_fac_3:SexM              -0.028889432 0.129506910 -0.2230725
## Replicate_fac_4:SexM              -0.330657951 0.131192842 -2.5203963
## factor(Tank)_A                     0.000000000          NA         NA
## factor(Tank)_B                    -0.085723339 0.048647254 -1.7621414
## scale(SampleOrder, scale = FALSE) -0.002896062 0.005171891 -0.5599619
## scale(Mass)                        0.061694991 0.041988385  1.4693347
## SexM                               0.897938354 0.114328804  7.8539993
## Replicate_fac_1                    0.000000000          NA         NA
## Replicate_fac_2                    0.475287662 0.087911209  5.4064512
## Replicate_fac_3                    0.329829938 0.088567297  3.7240601
## Replicate_fac_4                    0.435613988 0.089652689  4.8589060
## (Intercept)                        1.308361703 0.073195477 17.8748983
```

Conjugated cortisol

```
asr_cortconj_fac_rr2 <- asreml(log(Cort_conj_ng) ~ Replicate_fac + SexM +
                          scale(Mass) +
                          scale(SampleOrder, scale = FALSE) +
                            Tank,
                        random =~ str(~ID + ID:Replicate_cen, ~us(2):id(32)),
                        data = df_hormones)
```

```r
wald(asr_cortconj_fac_rr2, denDF = "numeric", ssType = "conditional")
```

```
## ASReml: Wed May 30 12:56:14 2018
##
##      LogLik         S2      DF      wall     cpu
##      11.8420      0.1728   120  12:56:14     0.0
##      11.8420      0.1728   120  12:56:14     0.0
##      11.8420      0.1728   120  12:56:14     0.0
##      11.8420      0.1728   120  12:56:14     0.0
##
## Finished on: Wed May 30 12:56:14 2018
##
## LogLikelihood Converged
```

```
## $Wald
##                                   Df denDF   F.inc  F.con Margin
## (Intercept)                        1  26.9 292.100 94.660
## Replicate_fac                      3  64.3   3.309  3.309      A
## SexM                               1  27.1  35.490  1.018      A
## scale(Mass)                        1  27.2  29.280 29.260      A
## scale(SampleOrder, scale = FALSE)  1 115.6   1.126  1.105      A
## Tank                               1  26.9   2.415  2.415      A
##                                             Pr
## (Intercept)                       2.634023e-10
## Replicate_fac                     2.552063e-02
## SexM                              3.219103e-01
## scale(Mass)                       9.908595e-06
## scale(SampleOrder, scale = FALSE) 2.952616e-01
## Tank                              1.318214e-01
##
## $stratumVariances
## NULL
```

```r
summary(asr_cortconj_fac_rr2, all = TRUE)$coef.fixed
```

```
##                                      solution   std error    z ratio
## Tank_A                             0.00000000          NA         NA
## Tank_B                            -0.18556304 0.119396578 -1.5541738
## scale(SampleOrder, scale = FALSE)  0.01033814 0.009832505  1.0514251
## scale(Mass)                        0.55671811 0.102917099  5.4093840
## SexM                               0.20729531 0.205445459  1.0090041
## Replicate_fac_1                    0.00000000          NA         NA
## Replicate_fac_2                   -0.03541668 0.105467951 -0.3358052
## Replicate_fac_3                    0.27660778 0.109933181  2.5161446
## Replicate_fac_4                    0.10497956 0.116997138  0.8972832
## (Intercept)                       -1.12496337 0.146146515 -7.6975039
```

Conjugated 11KT

Note that we use this constraint in the data to exclude a single value (not worth creating separate data frame as that row is included for the other response variables).

```r
asr_ktconj_fac_rr3 <- asreml(log(KT_conj_pg) ~ Replicate_fac + SexM +
                          scale(Mass) +
                          scale(SampleOrder, scale = FALSE) +
```

```r
                            Tank,
                        random =~ ID,
                        data = df_hormones[df_hormones$KT_conj_pg < 2000,])
```

```r
wald(asr_ktconj_fac_rr3, denDF = "numeric", ssType = "conditional")
```

```
## ASReml: Wed May 30 12:56:14 2018
##
##      LogLik         S2      DF      wall     cpu
##      -6.2805      0.2535   119  12:56:14     0.0
##      -6.2805      0.2535   119  12:56:14     0.0
##      -6.2805      0.2535   119  12:56:14     0.0
##      -6.2805      0.2535   119  12:56:14     0.0
##
## Finished on: Wed May 30 12:56:14 2018
##
## LogLikelihood Converged
```

```
## $Wald
##                                    Df denDF    F.inc   F.con Margin
## (Intercept)                         1  27.3 1355.000 217.100
## Replicate_fac                       3  91.1    9.274   9.770      A
## SexM                                1  27.4   62.090  32.900      A
## scale(Mass)                         1  27.4    2.384   1.873      A
## scale(SampleOrder, scale = FALSE)   1 118.0   10.200  10.190      A
## Tank                                1  27.4    1.274   1.274      A
##                                              Pr
## (Intercept)                        1.584632e-14
## Replicate_fac                      1.186967e-05
## SexM                               4.061699e-06
## scale(Mass)                        1.822498e-01
## scale(SampleOrder, scale = FALSE) 1.807885e-03
## Tank                               2.687784e-01
##
## $stratumVariances
##                  df  Variance      ID R!variance
## ID          28.08777 0.6044090 3.84049          1
## R!variance 90.91223 0.2534544 0.00000          1
```

```r
summary(asr_ktconj_fac_rr3, all = TRUE)$coef.fixed
```

```
##                                       solution  std error   z ratio
## Tank_A                              0.00000000         NA        NA
## Tank_B                             -0.15819579 0.14014426 -1.128807
## scale(SampleOrder, scale = FALSE)  0.03685801 0.01154505  3.192538
## scale(Mass)                         0.16490416 0.12049595  1.368545
## SexM                                1.38098976 0.24077323  5.735645
## Replicate_fac_1                     0.00000000         NA        NA
## Replicate_fac_2                     0.13150066 0.12586065  1.044812
## Replicate_fac_3                     0.64611219 0.12706679  5.084823
## Replicate_fac_4                     0.17621091 0.12586065  1.400048
## (Intercept)                         1.76418585 0.16303709 10.820764
```

Among-individual variance

Reaction norm models

For each response trait in turn we tested for among-individual variance within the reaction norm framework. To test for repeatable differences in average hormone levels (i.e., among-individual variance in reaction norm intercept) across all four repeats we compare the following models (with fixed effects as in the simplified models above):

- No random effects
- A random effect of individual ID (testing for consistent individual differences)
- Random effects of ID and the interaction between ID and sampling repeat (as a continuous covariate), and their covariance

We compared nested models using likelihood ratio tests (LRTs), in which we assume that twice the difference in model log-likelihoods conforms to a chi-square distribution where the degrees of freedom are set by the number of additional parameters in the more complex model.

Free cortisol

```
asr_cort_rxn_null <- asreml(log(Cort_ng) ~ Replicate_fac + SexM +
                               scale(Mass) +
                               scale(SampleOrder, scale = FALSE) +
                               Tank,
                             data = df_hormones)


asr_cort_rxn_RI <- asreml(log(Cort_ng) ~ Replicate_fac + SexM +
                             scale(Mass) +
                             scale(SampleOrder, scale = FALSE) +
                             Tank,
                           random =~ ID,
                           data = df_hormones)


asr_cort_rxn_RS <- asreml(log(Cort_ng) ~ Replicate_fac + SexM +
                             scale(Mass) +
                             scale(SampleOrder, scale = FALSE) +
                             Tank,
                           random =~ str(~ID + ID:Replicate_cen, ~us(2):id(32)),
                           data = df_hormones)
```

Test for among-individual differences in average cortisol level:

```
# Chi-square test statistic
2*(asr_cort_rxn_RI$loglik - asr_cort_rxn_null$loglik)
```

```
## [1] 3.108377
```

```
# P
0.5*pchisq(2*(asr_cort_rxn_RI$loglik - asr_cort_rxn_null$loglik),1,lower.tail = FALSE)
```

```
## [1] 0.03894527
```

Test for individual differences in habituation rate:

```
# Chi-square test statistic
2*(asr_cort_rxn_RS$loglik - asr_cort_rxn_RI$loglik)
```

```
## [1] 4.125288
```

```
# P
pchisq(2*(asr_cort_rxn_RS$loglik - asr_cort_rxn_RI$loglik),2,lower.tail = FALSE)
```

```
## [1] 0.1271175
```

Free 11KT

```
asr_11kt_rxn_null <- asreml(log(KT_pg) ~ Replicate_fac * SexM +
                              scale(Mass) +
                              scale(SampleOrder, scale = FALSE) +
                              Tank,
                            data = df_hormones)


asr_11kt_rxn_RI <- asreml(log(KT_pg) ~ Replicate_fac * SexM +
                            scale(Mass) +
                            scale(SampleOrder, scale = FALSE) +
                            Tank,
                          random =~ ID,
                          data = df_hormones)


asr_11kt_rxn_RS <- asreml(log(KT_pg) ~ Replicate_fac * SexM +
                            scale(Mass) +
                            scale(SampleOrder, scale = FALSE) +
                            Tank,
                          random =~ str(~ID + ID:Replicate_cen, ~us(2):id(32)),
                          data = df_hormones,
                          maxiter = 200)
```

Test for among-individual differences in average 11KT level:

```
# Chi-square test statistic
2*(asr_11kt_rxn_RI$loglik - asr_11kt_rxn_null$loglik)
```

```
## [1] 0.1364464
```

```
# P
0.5*pchisq(2*(asr_11kt_rxn_RI$loglik - asr_11kt_rxn_null$loglik),1,lower.tail = FALSE)
```

```
## [1] 0.3559198
```

Test for individual differences in habituation rate:

```
# Chi-square test statistic
2*(asr_11kt_rxn_RS$loglik - asr_11kt_rxn_RI$loglik)
```

```
## [1] -0.179118
```

```
# P
pchisq(2*(asr_11kt_rxn_RS$loglik - asr_11kt_rxn_RI$loglik),2,lower.tail = FALSE)
```

```
## [1] 1
```

Character state models

For each response variable we formulated a multivariate (4-'trait') model to test hypotheses about variance in – and covariance among – the four repeat-specific observations. Rather than using the raw data, we estimated (co)variances conditional on the fixed effects of sex, size, tank and order (as described above).

We fitted a series of nested models to test hypotheses about the structure of individual variation:

- Model 1 estimates no covariances, and constrains the repeat-specific variances to be equal.
- Model 2 allows these variances to differ.
- Model 3 extends model 2 by also estimating all covariances.

We compared nested models using likelihood ratio tests (LRTs), in which we assume that twice the difference in model log-likelihoods conforms to a chi-square distribution where the degrees of freedom are set by the number of additional parameters in the more complex model. Model 2 vs model 1 therefore tests whether phenotypic variance (conditional on fixed effects) changes significantly across repeats, and model 3 vs model 2 tests for the existence of significant within-individual covariance structure (i.e., that some degree of repeatability exists). Model 3 estimates the within-individual covariance-correlation matrix (conditional on fixed effects), which we used as the basis for a parametric bootstrap method (described in Boulton et al. 2014; Houslay et al. 2017) to generate approximate 95% CI on all parameters.

Note that fitting a multivariate structure to data in 'long' rather than 'wide' format means that here we group by identity, but restrict the residual variance to effectively 0 (as each individual is only measured once in each 'repeat').

Free cortisol

```r
# Set up a model to get starting values so that we can set residual variation to (effectively) 0
asr_cort_fac_CS_sv <- asreml(log(Cort_ng) ~ Replicate_fac + SexM +
                               scale(Mass) +
                               scale(SampleOrder, scale = FALSE) +
                               Tank,
                             random =~ us(Replicate_fac):ID,
                             data = df_hormones,
                             start.values = TRUE)

# Pull out SVs, fix residual to very close to 0
cort_gt <- asr_cort_fac_CS_sv$gammas.table
cort_gt[11,2] <- 1e-8
cort_gt[11,3] <- "F"
# We then use these modified starting values in the residual parameters for future models

# Now run the models with this constraint

# idv: single variance for random effect, no covariances
asr_cort_fac_CS_idv <- asreml(log(Cort_ng) ~ Replicate_fac + SexM +
                                scale(Mass) +
                                scale(SampleOrder, scale = FALSE) +
                                Tank,
                              random =~ idv(Replicate_fac):ID,
                              data = df_hormones,
                              #G.param= cort_gt,
                              R.param= cort_gt)

# idh: variances can differ, no covariances
asr_cort_fac_CS_idh <- asreml(log(Cort_ng) ~ Replicate_fac + SexM +
```

```
                                  scale(Mass) +
                                  scale(SampleOrder, scale = FALSE) +
                                  Tank,
                                random =~ idh(Replicate_fac):ID,
                                data = df_hormones,
                                #G.param= cort_gt,
                                R.param= cort_gt,
                                maxiter = 200)

# us: estimate all variances and covariances
asr_cort_fac_CS_us <- asreml(log(Cort_ng) ~ Replicate_fac + SexM +
                                 scale(Mass) +
                                 scale(SampleOrder, scale = FALSE) +
                                 Tank,
                               random =~ us(Replicate_fac):ID,
                               data = df_hormones,
                               #G.param= cort_gt,
                               R.param= cort_gt)
```

```
# Are 4 variances better than 1?
pchisq(2*(asr_cort_fac_CS_idh$loglik - asr_cort_fac_CS_idv$loglik),3,lower.tail = FALSE)
```

```
## [1] 0.03173446
```

```
# Is there significant covariance structure?
pchisq(2*(asr_cort_fac_CS_us$loglik - asr_cort_fac_CS_idh$loglik),6,lower.tail = FALSE)
```

```
## [1] 0.0292734
```

```
boot_cort_free <- bootRepMat(asr_cort_fac_CS_us, 4)

out_cort_free <- getOutputMat_Rep(asr_cort_fac_CS_us, 4)

colnames(out_cort_free) <- c("Repeat 1", "Repeat 2", "Repeat 3", "Repeat 4")
rownames(out_cort_free) <- c("Repeat 1", "Repeat 2", "Repeat 3", "Repeat 4")

kable(out_cort_free)
```

	Repeat 1	Repeat 2	Repeat 3	Repeat 4
Repeat 1	0.127 (0.059,0.191)	0.121 (-0.498,0.33)	0.345 (-0.067,0.476)	0.141 (-0.476,0.346)
Repeat 2	0.018 (-0.035,0.076)	0.182 (0.085,0.28)	0.34 (-0.069,0.496)	-0.239 (-1.289,0.085)
Repeat 3	0.06 (-0.005,0.124)	0.071 (-0.007,0.157)	0.237 (0.113,0.357)	0.265 (-0.204,0.425)
Repeat 4	0.03 (-0.047,0.111)	-0.062 (-0.155,0.033)	0.078 (-0.028,0.186)	0.364 (0.168,0.537)

Free 11KT

```
# Set up a model to get starting values so that we can set residual variation to (effectively) 0
asr_KT_fac_CS_sv <- asreml(log(KT_pg) ~ Replicate_fac * SexM +
                                 scale(Mass) +
                                 scale(SampleOrder, scale = FALSE) +
                                 Tank,
                               random =~ us(Replicate_fac):ID,
                               data = df_hormones,
                               start.values = TRUE)
```

```r
# Pull out SVs, fix residual to very close to 0
KT_gt <- asr_KT_fac_CS_sv$gammas.table
KT_gt[11,2] <- 1e-8
KT_gt[11,3] <- "F"

# Now run the models with this constraint

# idv: single variance for random effect, no covariances
asr_KT_fac_CS_idv <- asreml(log(KT_pg) ~ Replicate_fac * SexM +
                                scale(Mass) +
                                scale(SampleOrder, scale = FALSE) +
                                Tank,
                              random =~ idv(Replicate_fac):ID,
                              data = df_hormones,
                              #G.param= KT_gt,
                              R.param= KT_gt)

# idh: variances can differ, no covariances
asr_KT_fac_CS_idh <- asreml(log(KT_pg) ~ Replicate_fac * SexM +
                                scale(Mass) +
                                scale(SampleOrder, scale = FALSE) +
                                Tank,
                              random =~ idh(Replicate_fac):ID,
                              data = df_hormones,
                              #G.param= KT_gt,
                              R.param= KT_gt,
                              maxiter = 200)

# us: estimate all variances and covariances
asr_KT_fac_CS_us <- asreml(log(KT_pg) ~ Replicate_fac * SexM +
                               scale(Mass) +
                               scale(SampleOrder, scale = FALSE) +
                               Tank,
                             random =~ us(Replicate_fac):ID,
                             data = df_hormones,
                             #G.param= KT_gt,
                             R.param= KT_gt)
```

```r
# Are 4 variances better than 1?
pchisq(2*(asr_KT_fac_CS_idh$loglik - asr_KT_fac_CS_idv$loglik),3,lower.tail = FALSE)
```

```
## [1] 0.8910099
```

```r
# Is there significant covariance structure?
pchisq(2*(asr_KT_fac_CS_us$loglik - asr_KT_fac_CS_idh$loglik),6,lower.tail = FALSE)
```

```
## [1] 0.795947
```

```r
boot_KT_free <- bootRepMat(asr_KT_fac_CS_us, 4)

out_KT_free <- getOutputMat_Rep(asr_KT_fac_CS_us, 4)

colnames(out_KT_free) <- c("Repeat 1", "Repeat 2", "Repeat 3", "Repeat 4")
rownames(out_KT_free) <- c("Repeat 1", "Repeat 2", "Repeat 3", "Repeat 4")
```

```r
kable(out_KT_free)
```

	Repeat 1	Repeat 2	Repeat 3	Repeat 4
Repeat 1	0.076 (0.035,0.116)	-0.184 (-1.13,0.13)	-0.017 (-0.793,0.229)	0.197 (-0.35,0.386)
Repeat 2	-0.013 (-0.037,0.014)	0.063 (0.031,0.095)	0.143 (-0.493,0.333)	-0.048 (-0.785,0.223)
Repeat 3	-0.001 (-0.029,0.026)	0.01 (-0.017,0.035)	0.076 (0.037,0.114)	0.103 (-0.536,0.31)
Repeat 4	0.013 (-0.011,0.04)	-0.003 (-0.024,0.021)	0.007 (-0.018,0.032)	0.059 (0.03,0.091)
